# New Quinoline-Based Heterocycles as Anticancer Agents Targeting Bcl-2

**DOI:** 10.3390/molecules24071274

**Published:** 2019-04-02

**Authors:** Rania Hamdy, Samia A. Elseginy, Noha I. Ziedan, Arwyn T. Jones, Andrew D. Westwell

**Affiliations:** 1 School of Pharmacy and Pharmaceutical Sciences, Cardiff University, Redwood Building, King Edward VII Avenue, Cardiff, Wales CF10 3NB, UK; rania_hamdy2000@yahoo.com (R.H.); samiaaliph@hotmail.com (S.A.E.); n.ziedan@chester.ac.uk (N.I.Z.); JonesAT@cardiff.ac.uk (A.T.J.); 2 Sharjah Institute for Medical Research, College of Pharmacy, University of Sharjah, P.O. Box 27272, Sharjah, UAE; 3 Faculty of Pharmacy, Zagazig University, Zagazig 445519, Egypt; 4 Green Chemistry Department, Chemical Industries Research Division, National Research Center, Dokki, Giza, P.O. Box 12622, Egypt; 5 School of Biochemistry, University of Bristol, University Walk, Bristol BS8 1TD, UK; 6 Department of Natural Sciences, University of Chester, Chester CH2 4NU, UK

**Keywords:** aromatic heterocycles, quinoline, oxadiazole, triazole, anticancer, Bcl-2 inhibitor, ELISA, molecular modelling, apoptosis

## Abstract

The Bcl-2 protein has been studied as an anticancer drug target in recent years, due to its gatekeeper role in resisting programmed cancer cell death (apoptosis), and the design of BH3 domain mimetics has led to the clinical approval of Venetoclax (ABT-199) for the treatment of chronic lymphocytic leukaemia. In this work we extend our previous studies on the discovery of indole-based heterocycles as Bcl-2 inhibitors, to the identification of quinolin-4-yl based oxadiazole and triazole analogues. Target compounds were readily synthesized via a common aryl-substituted quinolin-4-carbonyl-*N*-arylhydrazine-1-carbothioamide (**5a–b**) intermediate, through simple variation of the basic cyclisation conditions. Some of the quinoline-based oxadiazole analogues (e.g. compound **6i**) were found to exhibit sub-micromolar anti-proliferative activity in Bcl-2-expressing cancer cell lines, and sub-micromolar IC_50_ activity within a Bcl2-Bim peptide ELISA assay. The Bcl-2 targeted anticancer activity of **6i** was further rationalised via computational molecular modelling, offering possibilities to extend this work into the design of further potent and selective Bcl-2 inhibitory heteroaromatics with therapeutic potential.

## 1. Introduction

**Figure 1:**
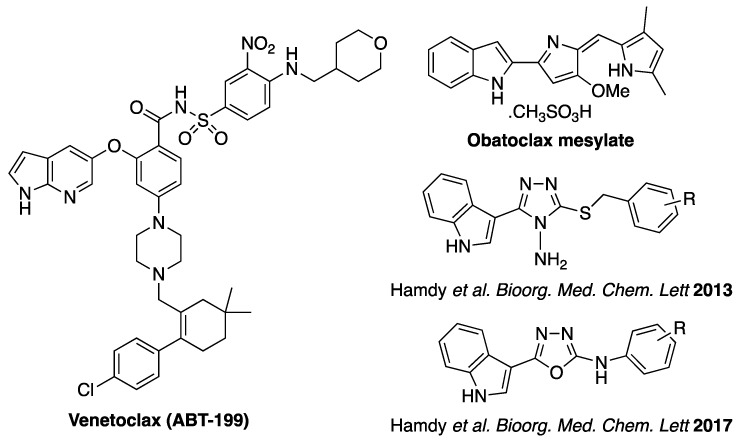
Bcl-2 small molecule inhibitors.

Resistance to apoptosis (programmed cell death) is an extensively studied cancer hallmark [1] that has inspired the development of targeted therapeutic approaches towards unlocking apoptotic signaling and selective induction of cancer cell death [2]. Bcl-2 is the archetypal member of a well-studied family of anti-apoptotic gatekeeper proteins, with Bcl-2 inhibition exploited in anticancer drug design [2]. Extensive drug development work over many years focused on Bcl-2 homology 3 (BH3) mimetics has recently led to the clinical approval of Venetoclax (ABT-199, Figure 1) for the treatment of chronic lymphocytic leukaemia in patients with a 17p deletion and at least one prior therapy [3]. Obatoclax mesylate (Figure 1) is a further example of an indole-based pan-Bcl-2 inhibitor that has been studied in cancer clinical trials (e.g. leukaemia and lymphoma) [4]. 

Our own work has led to the early stage design and discovery of indole-based heterocycles as new Bcl-2 inhibitors based on a molecular modelling and virtual screening approach to identify hit molecules. We have previously reported on series of 3-(benzylthio)-5-(indol-3-yl)-1,2,4-triazol-4-amines, 5-(indol-3-yl)-*N*-aryl-[1,3,4]-oxadiazol-2-amines and 5-(indol-3-yl)-2-[(2-nitrophenyl)amino][1,3,4]-oxadiazole as Bcl-2 inhibitory anticancer agents (Figure 1) [5,6,7]. In this study, we further explore structure-activity relationships around this heterocyclic framework and synthesise further novel nitrogen-containing aromatic heterocycles for characterisation and biological testing, focusing on the corresponding quinoline derivatives.

## 2. Results

### 2.1. Synthesis of Quinolinyl-N-Aryl-Oxadiazolamines and Related Triazole Derivatives as Bcl-2 Inhibitory Anticancer Agents

**Scheme 1:**
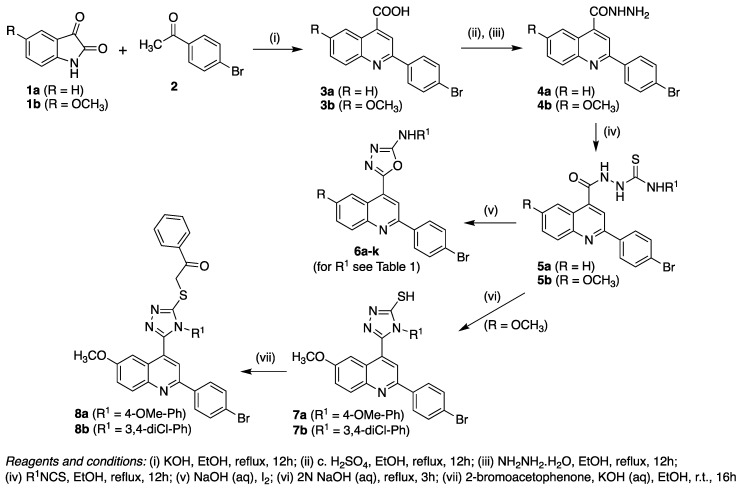
Synthesis of substituted quinolin-4-yl-*N*-aryl-oxadiazol-2-amines (**6a–k**) and quinolin-4-yl-benzoylmethylthiotriazole (**8a–b**) derivatives.

The synthetic route to target compounds is outlined in Scheme 1. Commercially available isatin compounds **1a–b** were first reacted with 4-bromoacetophenone (**2**) under basic conditions in refluxing ethanol to provide the intermediate 2-(4-bromophenyl)quinoline-4-carboxylic acids **3a–b** according to the standard Pfitzinger reaction protocol [8]. Following construction of the quinoline framework, the carboxylic acid function was first esterified then reacted with hydrazine hydrate to provide the corresponding hydrazide derivatives **4a–b**. Reaction of compounds **4a–b** with aryl isothiocyanates in ethanol under reflux gave the corresponding 2-(2-(4-bromophenyl)quinoline-4-carbonyl)-*N*-aryl-hydrazine-1-carbothioamide (acylthiosemicarbazide) intermediates (**5a–k**) in good yields (>60%) that were appropriately functionalised to form the desired oxadiazole-2-amine and triazole-2-thione target heterocycles. 

Careful control of the quinoline-4-carbonyl-*N*-aryl-hydrazine-1-carbothioamide basic cyclisation conditions led selectively to alternative heterocyclic cyclisation products. Treatment of compounds **5a–k** with aqueous sodium hydroxide in the presence of iodine as oxidant led to the *N*-aryl-1,3,4-oxadiazole products **6a–k** according to literature precedent [9], whereas refluxing in 2N sodium hydroxide produced the *N*-aryl-1,3,4-triazole-2-thiones **7a–b**. Intramolecular cyclodehydration of acrylthiosemicarbazides to give product 1,3,4-triazole-2-thione derivatives under basic conditions as is the case here, compared to product 1,3,4-thiadiazole-2-amine derivatives under acidic conditions, follows literature precedent [10,11,12]. The *N*-aryl-1,3,4-triazine-2-thiones (**7a–b**) were further S-alkylated using 2-bromoacetophenone under basic conditions to give the final products **8a–b**, containing a bulky S-substituent projected to sit within a hydrophobic pocket of the Bcl-2 binding site.

### 2.2. In Vitro Cancer Cell Line Testing for Anti-Proliferative Activity

Evaluation of anti-proliferative activity for the new quinolin-4-yl-*N*-aryloxadiazol-2-amines (**6a–k**) and quinolin-4-yl-benzoylmethylthiotriazoles (**8a–b**) was carried out in established MDA-MB-231 (metastatic breast) and HeLa (cervical) cancer cell lines using the MTT endpoint assay, according to methodology previously described by our group and others [5,6]. MDA-MB-231 and HeLa are Bcl2-expressing cancer cell lines where Bcl-2 inhibitory molecules have shown protein down-regulation following treatment [13,14]. Additionally, to test the effect of differing cellular Bcl-2 status, compounds **6a–k** and **8a–b** were evaluated for activity in the leukaemic cell lines KG1a (acute myelogenous leukaemia, Bcl-2 positive, [15]) and Jurkat (T-cell leukaemia, Bcl-2 negative, [16]). As previously described [5,6], for the studies in the leukaemic cell lines we used the CellTitreBlue® endpoint assay, appropriate for testing anti-proliferative activity in these non-adherent cells.

The results presented in Table 1 indicate some potent (sub-micromolar) IC_50_ activity, particularly for some of the oxadiazole-based compounds in the solid tumour cell lines MDA-MB-231 (**6b–c**, **6f–k)** and HeLa (**6c**, **6e–h**, **6j**). Potent (low to sub-micromolar) activity was also observed for a few oxadiazole compounds in the Bcl-2-expressing KG1a cell line (**6d–e**, **6i–j**). All compounds were found to be essentially inactive within the Bcl-2 negative Jurkat cell line. Amongst individual compounds with potent anti-proliferative activity across the three sensitive cell lines, compound **6j** is of particular note, having sub-micromolar IC_50_ values in these three cell lines. The two triazole-based analogues **8a** and **8b** were found to be inactive in all cell lines tested, and were not explored further.

### 2.3. Evaluation of Bcl-2 Binding via Enzyme-Linked Immunosorbent Assay (ELISA)

The ability of active compounds **6c**, **6d** and **6i** to compete with the pro-apoptotic BH3 (Bcl-2 homology domain 3)-binding Bim peptide was evaluated using an ELISA (enzyme-linked immunosorbent assay), according to our previously described protocol [5,6]. Compounds **6c**, **6d** and **6i** were chosen to represent compounds that were amongst the most potent tested across the cell lines (**6c** and **6i**) and a compound of intermediate potency (**6d**) to assess whether cellular potency in Bcl-2 expressing cell lines correlates with Bcl-2 binding activity. 

Briefly, biotinylated Bim peptide was attached to streptavidin-coated microtitre plates. The addition of mixtures of **6c**, **6d**, or **6i** at various concentrations and His-tagged Bcl-2 allowed competitive binding between test compounds and Bim peptide for His-tagged Bcl-2 binding. Addition of anti-His secondary antibody conjugated to HRP (horseradish peroxidase) enzyme was followed by the addition of *o*-phenylenediamine, which produces a colourimetric readout in the presence of peroxidase enzyme (monitored at 450 nm). Reduction of the signal by competitive binding of the test compound to Bcl-2 is then plotted against concentration to derive an IC_50_ for test compound inhibition of Bim-BH3 binding. The well-known Bcl-2 inhibitory natural product (-)-gossypol, which has been used in cancer clinical trials [17], was used as a positive control. The results of the ELISA assay are shown in Table 2.

The results of the ELISA study shown in Table 2 provide direct evidence for the ability of the new compounds to compete with Bim peptide for direct binding to Bcl-2. Notably compound **6d** with moderate anti-proliferative activity in Bcl-2 expressing cell lines was the least potent compound in the ELISA assay. In contrast, compound **6i** gave an IC_50_ value (0.15 μM) more potent than the positive control Bcl-2 inhibitor gossypol.

### 2.4. Molecular Modelling of Compound **6i** Interacting within the Bcl-2 Binding Pocket

**Figure 2:**
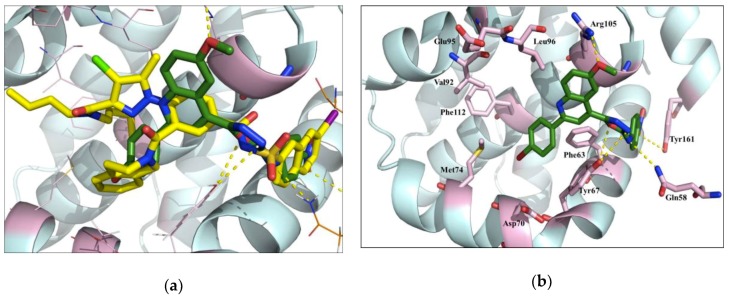
(**a**) Representation of the binding mode of compound **6i** (green stick) and native iodonaphthalene sulfonamide-based ligand (yellow stick) from the co-crystal structure with Bcl-2 (PDB code 4AQ3), showing the overlap in binding poses between the two ligands. (**b**) representative interactions of compound **6i** (dark green stick) with key residues (purple stick) of the Bcl-2 binding pocket. H-bonds represented as yellow dotted lines.

To gain insight into binding interactions at the molecular level, and to further validate our drug design and Bcl-2 binding (ELISA) results allowing future optimization of heterocyclic Bcl-2 inhibitors, we undertook computational molecular modeling work on hit compound **6i** within the Molecular Operating Environment (MOE) platform [18]. Our starting point was a published co-crystal structure (Protein Data Bank (PDB) code: 4AQ3) between a sulfonamide-based dual Bcl-2/Bcl-xL antagonist (*N,N*-dibutyl-4-chloro-1-[2-(3,4-dihydro-1H-isoquinolin-2-ylcarbonyl)-4-[(7-iodonaphthalene-2-yl)sulfonylcarbamoyl]phenyl]-5-methyl-pyrazole-3-carboxamide) and the Bcl-2 binding pocket [19]. The active site of Bcl-2 is formed of two hydrophobic pockets (G1 and G2) and a shallow linker. The interaction of the native sulfonamide ligand within the Bcl-2 binding site showed a number of key interactions, including: (i) a H-bond between the oxygen atom of the sulfonamide SO_2_ group and NH_2_ of Gln58; (ii) two H-bonds between the oxygen atom of sulfonamide SO_2_/NH and the OH of Tyr67. The iodonaphthalene moiety showed a face–face aromatic interaction with Tyr161. Additional hydrophobic interactions with Ala59, Phe63, Tyr161 constitute the hydrophobic site of Bcl-2. Furthermore, the isoquinoline moiety formed hydrophobic interactions with Phe63, Phe71 and Ala108, and the dibutylamine side chain showed hydrophobic interactions with Val92, Leu96, Phe112, and the hydrophobic part of Glu95 and Met97. Figure 2 shows this ligand binding in more detail (yellow stick model).

Our docking results for {5-[2-(4-bromophenyl)-6-methoxyquinolin-4-yl}-*N*-(4-nitrophenyl)-[1,3,4]oxadiazol-2-amine (**6i**; green stick model) in the Bcl-2 binding pocket indicate a number of similarities to the native co-crystallised sulfonamide ligand, showing five H-bonds with key Bcl-2 binding site residues. The following interactions are notable: (i) the oxygen atom of the NO_2_ group was a H-bond acceptor from the OH of Tyr161 (distance 3.4Å); (ii) the NH group bridging the oxadiazole and nitrophenyl formed H-bonds with the NH_2_ of Gln58 (distance 3.4Å); (iii) similarly to the co-crystalized ligand the oxadiazole moiety of **6i** formed two H-bonds with key residue Tyr67; (iv) the nitrogen and oxygen atoms of the oxadiazole formed two H-bonds with the OH of Tyr67 (distance 3.5Å and 3.3Å respectively); (v) the O atom of the OCH_3_ group was a H-bond acceptor with the NH_2_ of Arg105 (distance 2.6Å). Figure 2a shows the binding interactions of **6i** in more detail and binding modes between the two ligands.

Overall the quinolin-4-yl-*N*-aryl-[1,3,4]oxadiazol-2-amine (**6i**) showed a similar binding mode within the Bcl-2 pocket compared to the native sulfonamide ligand, noting that the nitrophenylamino-oxadiazole component of **6i** occupied the same position as the iodonaphthalene sulfonamide moiety of the native ligand (Figure 2a). Similarly the phenyl-quinoline component of compound **6i** occupied the same position as the phenyl-isoquinoline moiety of the native ligand within the hydrophobic pocket of Bcl-2. Figure 2b illustrates the key binding interactions for compound **6i** within the Bcl-2 binding pocket in more detail, with the nitrophenylamino-oxadiazole showing interactions with Gln58, Phe63, Tyr67 and Tyr161. Meanwhile the bromophenyl-quinoline moiety showed hydrophobic interactions with Val92, Leu96, and Phe112 residues, with the 6-methoxy group making an additional interaction with Arg105. Overall, it could be concluded that the quinoline-oxadiazole based hit compound **6i** makes key interactions with the Bcl-2 active site thereby contributing to Bcl-2 inhibitory activity.

## 3. Discussion and Conclusions

This new series of quinoline-based heterocycles further supports our concept that rationally designed Bcl-2 inhibitory aromatic heterocycles can effectively interfere with Bcl-2–Bim peptide binding and exhibit potent anti-proliferative activity against Bcl-2 expressing human cancer cell lines. Access to the target compounds was achieved via a straightforward and efficient synthetic route. In particular, our research demonstrated complete regiochemical control of cyclisation from a common 2-(2-(4-aryl)quinoline-4-carbonyl)-*N*-aryl-hydrazine-1-carbothioamide intermediate (**5a–k**) to form either quinolin-4-yl-*N*-aryl-oxadiazol-2-amines (**6a–k**) or quinolin-4-yl-benzylthiotriazoles (**8a–b**) by simple variation of the basic reaction conditions. From the compounds active in Bcl-2 expressing cancer cell lines and selected for further study, compound **6i** exhibited potent inhibitory activity in the Bcl-2-Bim peptide ELISA assay. On the other hand, compound **6c**, overall one of the most potent compounds against Bcl-2 expressing cell lines, gave a moderate ELISA IC**_50_** value. This mismatch between cellular and target-based activity is likely indicative of additional molecular targets for these molecules as yet undiscovered, as would be expected to be the case for small heterocyclic molecules of this type. The quinoline-oxadiazole based compound **6i** (R^1^ = 4-nitrophenyl) in particular was found to exhibit potency within Bcl-2 expressing cancer cells and the ELISA assay and will be the subject of further investigation.

## 4. Experimental

### 4.1. General Experimental Details—Chemistry

Nuclear magnetic resonance (NMR) spectra were recorded on a Bruker Avance 500 MHz instrument, and chemical shifts are given in ppm relative to Me_4_Si with coupling constants (*J* values) in Hz. Mass spectrometry was run in electrospray positive ionization mode (Bruker MicroTof instrument, Coventry, UK). Elemental analysis (% CHN) was run by combustion analysis through an outsourced service (Medac Ltd, Surrey, UK). Commercial compounds were used as received; 2-(4-bromophenyl)quinoline-4-carboxylic acid (**3a**) [20] and 2-(4-bromophenyl)-6-methoxyquinoline-4-carboxylic acid (**3b**) [21] were accessed via the standard Pfitzinger reaction protocol [8].

### 4.2. Preparation of 2-(4-Bromophenyl)Quinoline-4-Carboxylic Acid Hydrazides (**4a**–**b**)

A mixture of the appropriate 2-(4-bromophenyl)quinoline-4-carboxylic acid (**3a–b**) (10 mmol), absolute ethanol (20 mL) and concentrated sulfuric acid (2 mL) was heated under reflux for 12 h. Excess ethanol was removed under reduced pressure and the resulting oil was rendered alkaline using aqueous sodium bicarbonate. The aqueous layer was extracted with dichloromethane (2 × 50 mL) and the combined organic extracts were dried over anhydrous sodium sulfate and concentrated under reduced pressure. The intermediate crude ester product was re-dissolved in ethanol (10 mL) and 98% hydrazine hydrate (10 mL) added. The solution was heated under reflux for a further 12 h and then allowed to cool to room temperature. The precipitate that formed was collected by filtration, washed with water (3 × 10 mL) to remove excess hydrazine hydrate, and dried *in vacuo* to give the intermediate quinoline carboxylic hydrazides (**4a–b**) in 75%–76% overall yield.

*2-(4-Bromophenyl)quinoline-4-carboxylic acid hydrazide* (**4a**). Yield 75%, m.p. 246–248 °C. ^1^H-NMR (DMSO-*d*_6_ ): 4.7 (s, 2H, NH_2_), 7.65 (t, 1H, ArCH), 7.79 (d, 2H, *J* = 8.9, ArCH), 7.87 (t, 1H, *J* = 7.5, ArCH), 8.13 (d, 2H, *J* = 2.9, ArCH), 8.24 (d, 1H, *J* = 5.7, CH aromatic), 8.27 (d, 2H, *J* = 8.9, ArCH), 10.02 (s, 1H, NH).

*2-(4-Bromophenyl)-6-methoxyquinoline-4-carboxylic acid hydrazide* (**4b**). Yield 76%, m.p. 266–268 °C. ^1^H-NMR (DMSO-*d*_6_ ): 3.9 (s, 3H, OCH_3_), 4.7 (s, 2H, NH_2_), 7.5 (d, 1H, *J* = 7.5, ArCH), 7.68 (d, 1H, *J* = 2.4, ArCH), 7.70 (d, 2H, *J* = 7.5, ArCH), 8.07 (d, 1H, *J* = 7.5, ArCH), 8.13 (s, 1H, ArCH), 8.27 (d, 2H, *J* = 7.5, ArCH), 10.09 (s, 1H, NH).

### 4.3. Preparation of 2-(2-(4-Bromophenyl)Quinoline-4-Carbonyl)-N-Arylhydrazine-1-Carbothioamides (**5a**–**k**)

To a solution of quinoline-4-carboxylic acid hydrazide (**4a–b**, 1 mmol) in absolute ethanol (20 mL) was added a solution of substituted phenylisothiocyanate (1 mmol) in ethanol (10 mL) with continuous stirring. The reaction mixture was heated under reflux for 12 h. After cooling to room temperature, the precipitate formed was collected by filtration, and washed with ice-cold ethanol (5 mL) to give the corresponding quinoline-4-carbonyl-*N*-arylhydrazine-1-carbothioamide (**5a–k**) which was used in the next step without further purification.

*2-(2-(4-Bromophenyl)quinoline-4-carbonyl)-N-(4-chlorophenyl)hydrazine-1-carbothioamide* (**5a**). Yield 74%, m.p. 247–249 °C. ^1^H-NMR (DMSO-*d*_6_): 7.45–7.62 (m, 4H, ArCH), 7.67 (t, 1H, *J* = 7.5, ArCH), 7.80 (d, 1H, *J* = 9.2, ArCH), 7.90 (d, 1H, *J* = 8.6, ArCH), 8.14 (d, 1H, *J* = 8.5, ArCH), 8.25 (d, 1H, *J* = 9.2, ArCH), 8.34 (d, 2H, *J* = 7.5, ArCH), 8.42 (d, 2H, *J*= 2.5, ArCH), 10.02 (s, 2H, NH), 10.95 (s, 1H, NH).

*2-(2-(4-Bromophenyl)quinoline-4-carbonyl)-N-(3,4-dichlorophenyl)hydrazine-1-carbothioamide* (**5b**). Yield 65%, m.p. 265–267 °C. ^1^H-NMR (DMSO-*d*_6_): 7.60 (m, 3H, ArCH), 7.77 (d, 1H, *J* = 8.5, ArCH), 7.85 (d, 2H, *J* = 8.5, ArCH), 8.09 (d, 1H, *J* = 7.5, ArCH), 8.19 (m, 1H, ArCH), 8.29 (m, 2H, ArCH), 8.42 (d, 2H, *J* = 7.5, ArCH), 10.18 (s, 1H, NH), 10.82 (s, 1H, NH), 11.28 (s, 1H, NH).

*2-(2-(4-Bromophenyl)quinoline-4-carbonyl)-N-(4-methylphenyl)hydrazine-1-carbothioamide* (**5c**). Yield 69%, m.p. 245–247 °C. ^1^H-NMR (DMSO-*d*_6_): 2.31 (s, 3H, CH_3_), 7.18 (d, 2H, *J* = 8.5, ArCH), 7.35 (s, 2H, ArCH), 7.70 (m, 1H, ArCH), 7.85 (m, 3H, ArCH), 8.16 (d, 1H, *J* = 8.5, ArCH), 8.26 (d, 2H, *J* = 8.47, ArCH), 8.41 (d, 1H, *J* = 8.5, ArCH), 8.46 (s, 1H, ArCH), 9.83 (s, 2H, NH), 10.86 (s, 1H, NH).

*2-(2-(4-Bromophenyl)quinoline-4-carbonyl)-N-(4-nitrophenyl)hydrazine-1-carbothioamide* (**5d**). Yield 75%, m.p. 255–257 °C. ^1^H-NMR (DMSO-*d*_6_): 7.72 (m, 1H, ArCH), 7.75 (d, 3H, *J* = 8.5, ArCH), 7.86 (m, 2H, ArCH), 8.15 (d, 1H, *J* = 8.5, ArCH), 8.33 (m, 4H, ArCH), 8.49 (d, 2H, *J* = 3.5, ArCH), 10.35 (s, 2H, NH), 11.09 (s, 1H, NH).

*2-(2-(4-Bromophenyl)quinoline-4-carbonyl)-N-(4-methoxyphenyl)hydrazine-1-carbothioamide* (**5e**). Yield 61%, m.p. 216–218 °C. ^1^H-NMR (DMSO-*d*_6_): 3.79 (s, 3H, OCH_3_), 6.85 (d, 2H, *J* = 7.8, ArCH), 7.35 (d, 2H, *J* = 2.5, ArCH), 7.70 (m, 1H, ArCH), 7.82 (d, 2H, *J* = 7.8, ArCH), 7.90 (d, 1H, *J* = 6.5, ArCH), 8.17 (d, 1H, *J* = 8.5, ArCH), 8.26 (d, 2H, *J* = 7.8, ArCH), 8.40 (d, 2H, *J* = 2.5, ArCH), 9.80 (s, 2H, NH), 10.45 (s, 1H, NH).

*2-(2-(4-Bromophenyl)-6-methoxyquinoline-4-carbonyl)-N-(4-chlorophenyl)hydrazine-1-carbothioamide* (**5f**). Yield 75%, m.p. 186–188 °C. ^1^H-NMR (DMSO-*d*_6_): 3.9 (s, 3H, OCH_3_), 7.48 (m, 5H, ArCH), 7.70 (d, 3H, *J* = 8.9, ArCH), 8.04 (d, 1H, *J* = 8.9, ArCH), 8.21 (d, 3H, *J* = 8.8, ArCH), 10.2 (s, 2H, NH), 10.89 (s, 1H, NH).

*2-(2-(4-Bromophenyl)-6-methoxyquinoline-4-carbonyl)-N-(3,4-dichlorophenyl)hydrazine-1-carbothioamide* (**5g**). Yield 73%, m.p. 199–201 °C. ^1^H-NMR (DMSO-*d*_6_): 3.9 (s, 3H, OCH_3_), 7.51 (m, 4H, ArCH), 7.56 (m, 2H, ArCH), 7.75 (d, 2H, *J* = 8.5, ArCH), 8.09 (d, 1H, *J* = 7.5, ArCH), 8.19 (d, 2H, *J* = 8.5, ArCH), 10.13 (s, 2H, NH), 11.28 (s, 1H, NH).

*2-(2-(4-Bromophenyl)-6-methoxyquinoline-4-carbonyl)-N-(4-methylphenyl)hydrazine-1-carbothioamide* (**5h**). Yield 65%, m.p. 209–211 °C. ^1^H-NMR (DMSO-*d*_6_): 2.30 (s, 3H, CH_3_), 3.90 (s, 3H, OCH_3_), 7.19 (d, 2H, *J* = 8.8, ArCH), 7.35 (s, 2H, ArCH), 7.53 (d, 2H, *J* = 7.2, ArCH), 7.69 (d, 3H, *J* = 7.5, ArCH), 8.03 (d, 1H, *J* = 7.5, ArCH), 8.23 (d, 2H, *J* = 7.5, ArCH), 9.83 (s, 2H, NH), 10.85 (s, 1H, NH).

*2-(2-(4-Bromophenyl)-6-methoxyquinoline-4-carbonyl)-N-(4-nitrophenyl)hydrazine-1-carbothioamide* (**5i**). Yield 71%, m.p. 175–177 °C. ^1^H-NMR (DMSO-*d*_6_): 3.90 (s, 3H, OCH_3_), 7.51 (d, 1H, *J* = 7.8, ArCH), 7.82 (d, 3H, *J* = 6.3, ArCH), 7.95 (s, 2H, ArCH), 8.10 (d, 1H, *J* = 9.0, ArCH), 8.30 (m, 5H, ArCH), 10.35 (s, 2H, ArCH), 11.09 (s, 1H, NH).

*2-(2-(4-Bromophenyl)-6-methoxyquinoline-4-carbonyl)-N-(4-methoxyphenyl)hydrazine-1-carbothioamide* (**5j**). Yield 70%, m.p. 207–209 °C. ^1^H-NMR (DMSO-*d*_6_): 3.75 (s, 3H, OCH_3_), 3.90 (s, 3H, OCH_3_), 6.90 (d, 2H, *J* = 7.5, ArCH), 7.37 (m, 2H, ArCH), 7.55 (d, 1H, *J* = 6.9, ArCH), 7.80 (d, 3H, *J* = 7.5, ArCH), 8.10 (d, 1H, *J* = 7.5, ArCH), 8.25 (d, 2H, *J* = 7.5, ArCH), 8.45 (s, 1H, ArCH), 9.80 (s, 2H, NH), 10.09 (s, 1H, NH). *m*/*z* (ES^+^) 537.04 (M^+^).

*2-(2-(4-Bromophenyl)-6-methoxyquinoline-4-carbonyl)-N-(4-fluorophenyl)hydrazine-1-carbothioamide* (**5k**). Yield 60%, m.p. 183–185 °C. ^1^H-NMR (DMSO-*d*_6_): 3.90 (s, 3H, OCH_3_), 7.19 (m, 2H, ArCH), 7.45 (m, 3H, ArCH), 7.76 (d, 3H, *J* = 7.9, ArCH), 8.12 (d, 1H, *J* = 8.6, ArCH), 8.23 (d, 2H, *J* = 7.6, ArCH), 8.43 (s, 1H, ArCH), 9.98 (s, 2H, NH), 10.89 (s, 1H, NH).

### 4.4. Preparation of {5-[2-(4-Bromophenyl)-Quinolin-4-yl}-N-Aryl-[1,3,4]Oxadiazol-2-Amines (**6a-k**)

A suspension of quinoline-4-carbonyl-*N*-arylhydrazine-1-carbothioamide (**5a–k**, 1 mmol) in 2N sodium hydroxide (10 mL) was heated under reflux for 3h. After cooling to room temperature, the crude product precipitate was collected by filtration. Recrystallisation from ethanol gave the corresponding pure quinolin-4-yl-*N*-aryl-[1,3,4]oxadiazol-2-amine (**6a-k**) in 40%–78% isolated yield.

*{5-[2-(4-Bromophenyl)quinolin-4-yl}-N-(4-chlorophenyl)-[1,3,4]oxadiazol-2-amine* (**6a**). Yield 76%, m.p. 254–257 °C. ^1^H-NMR (DMSO-*d*_6_): 7.49 (d, 2H, *J* = 6.9, ArCH), 7.72 (d, 2H, *J* = 7.2, ArCH), 7.83 (d, 3H, *J* = 7.2, ArCH), 7.95 (m, 1H, ArCH), 8.25 (m, 3H, ArCH), 8.5 (s, 1H, ArCH), 9.12 (d, 1H, *J* = 6.9, ArCH), 11.20 (s, 1H, NH). *m*/*z* (ES^+^) 478.10 (M^+^). Anal. (C_23_H_14_BrClN_4_O): % CHN required: C 57.82, H 2.95, N 11.73; found C 57.82, H 2.82, N 11.74.

*{5-[2-(4-Bromophenyl)quinolin-4-yl}-N-(3,4-dichlorophenyl)-[1,3,4]oxadiazol-2-amine* (**6b**). Yield 78%, m.p. 264–266 °C. ^1^H-NMR (DMSO-*d*_6_): 7.61 (dd, 1H, *J* = 7.5, 2.9, ArCH), 7.68 (d, 1H, *J* = 9.3, ArCH), 7.83 (m, 2H, ArCH), 7.88 (s, 1H, *J* = 2.5, ArCH), 7.94 (t, 1H, *J* = 7.5, ArCH), 8.04 (d, 1H, *J* = 2.7, ArCH), 8.22 (s, 1H, ArCH), 8.24 (d, 2H, *J* = 8.3, ArCH), 8.47 (s, 1H, ArCH), 9.13 (d, 1H, *J* = 7.8, ArCH), 11.38 (s, 1H, NH). *m*/*z* (ES^+^) 512.25 (M^+^). Anal. (C_23_H_13_BrCl_2_N_4_O): % CHN required: C 53.93, H 2.56, N 10.94; found C 53.59, H 2.68, N 10.67.

*{5-[2-(4-Bromophenyl)quinolin-4-yl}-N-(4-methylphenyl)-[1,3,4]oxadiazol-2-amine* (**6c**). Yield 40%, m.p. 265–267 °C. ^1^H-NMR (DMSO-*d*_6_): 2.96 (s, 3H, CH_3_), 7.23 (d, 2H, *J* = 8.2, ArCH), 7.6 (d, 2H, *J* = 8.2, ArCH), 7.70 (d, 2H, *J* = 8.5, ArCH), 7.95 (m, 1H, ArCH), 8.21 (d, 1H, *J* = 8.2, ArCH), 8.31 (d, 2H, *J* = 8.2, ArCH), 8.35 (d, 1H, *J* = 8.5, ArCH), 8.46 (s, 1H, ArCH), 9.15 (d, 1H, *J* = 8.5, ArCH), 10.86 (s, 1H, NH). ^13^C-NMR (DMSO-*d*_6_): 20.32 (CH_3_), 116.85, 117.36, 122.16, 123.86, 125.85, 128.37, 129.00, 129.10, 129.56, 129.99, 130.76, 131.24, 132.04, 135.81, 137.11, 148.32, 154.72, 156.20, 160.28. HRMS *m*/*z* (ES^+^) required 457.0586 (M^+^+1), found 457.0589.

*{5-[2-(4-Bromophenyl)quinolin-4-yl}-N-(4-nitrophenyl)-[1,3,4]oxadiazol-2-amine* (**6d**). Yield 59%, m.p. 237–239 °C. ^1^H-NMR (DMSO-*d*_6_): 7.86 (d, 3H, *J* = 8.5, ArCH), 7.98 (m, 3H, ArCH), 8.27 (m, 3H, ArCH), 8.35 (d, 2H, *J* = 8.5, ArCH), 8.50 (s, 1H, ArCH), 9.15 (d, 1H, *J* = 7.5, ArCH), 11.84 (s, 1H, NH). *m*/*z* (ES^+^) 489.25 (M^+^). Anal. (C_23_H_14_BrN_5_O_3_): % CHN required: C 56.57, H 2.89, N 14.34; found C 56.59, H 2.79, N 14.37.

*{5-[2-(4-Bromophenyl)quinolin-4-yl}-N-(4-methoxyphenyl)-[1,3,4]oxadiazol-2-amine* (**6e**). Yield 65%, m.p. 229–231 °C. ^1^H-NMR (DMSO-*d*_6_): 3.79 (s, 3H, OCH_3_), 7.00 (d, 2H, *J* = 8.9, ArCH), 7.56 (d, 2H, *J* = 8.9, ArCH), 7.97 (m, 3H, ArCH), 7.92 (m, 1H, ArCH), 8.20 (m, 3H, ArCH), 8.50 (s, 1H, ArCH), 9.17 (d, 1H, *J* = 7.5, ArCH), 10.78 (s, 1H, NH). *m*/*z* (ES^+^) 474.05 (M^+^). Anal. (C_24_H_17_BrN_4_O_2_): % CHN required: C 60.90, H 3.62, N 11.84; found C 60.69, H 3.33, N 11.72.

*{5-[2-(4-Bromophenyl)-6-methoxyquinolin-4-yl}-N-(4-chlorophenyl)-[1,3,4]oxadiazol-2-amine* (**6f**). Yield 48%, m.p. 261–263 °C. ^1^H-NMR (DMSO-*d*_6_): 3.97 (s, 3H, OCH_3_), 7.41 (d, 2H, *J* = 6.9, ArCH), 7.54 (d, 1H, *J* = 7.6, ArCH), 7.63 (d, 2H, *J* = 6.9, ArCH), 7.75 (d, 2H, *J* = 7.45, ArCH), 8.15 (d, 1H, *J* = 8.5, ArCH), 8.24 (d, 2H, *J* = 8.5, ArCH), 8.40 (s, 1H, ArCH), 8.7 (d, 1H, *J* = 2.5, ArCH). *m*/*z* (ES^+^) 508.02 (M^+^). Anal. (C_24_H_16_BrClN_4_O_2_): % CHN required: C 56.77, H 3.18, N 11.03; found C 56.83, H 3.12, N 11.13.

*{5-[2-(4-Bromophenyl)-6-methoxyquinolin-4-yl}-N-(3,4-dichlorophenyl)-[1,3,4]oxadiazol-2-amine* (**6g**). Yield 59%, m.p. 227–229 °C. ^1^H-NMR (DMSO-*d*_6_): 3.95 (s, 3H, OCH_3_), 7.18 (d, 2H, *J* = 7.3, ArCH), 7.52 (d, 1H, *J* = 6.3, ArCH), 7.78 (d, 2H, *J* = 8.0, ArCH), 7.89 (d, 1H, *J* = 2.5, ArCH), 8.09 (d, 2H, *J* = 8.3, ArCH), 8.29 (d, 2H, *J* = 7.3, ArCH), 8.31 (s, 1H, NH), 8.90 (d, 1H, *J* = 2.3, ArCH). *m*/*z* (ES^+^) 541.98 (M^+^). Anal. (C_24_H_15_BrCl_2_N_4_O_2_): % CHN required: C 53.16, H 2.79, N 10.33; found C 52.92, H 2.86, N 10.12.

*{5-[2-(4-Bromophenyl)-6-methoxyquinolin-4-yl}-N-(4-methylphenyl)-[1,3,4]oxadiazol-2-amine* (**6h**). Yield 54%, m.p. 239–241 °C. ^1^H-NMR (DMSO-*d*_6_): 2.2 (s, 3H, CH_3_), 4.0 (s, 3H, OCH_3_), 7.20 (d, 2H, *J* = 8.3, ArCH), 7.62 (d, 3H, *J* = 8.7, ArCH), 7.75 (d, 2H, *J* = 9.1, ArCH), 8.09 (d, 1H, *J* = 9.1, ArCH), 8.2 (d, 2H, *J* = 8.3, ArCH), 8.40 (s, 1H, ArCH), 8.65 (d, 1H, *J* = 3.0, ArCH), 10.85 (s, 1H, NH). HRMS *m*/*z* (ES^+^) required 487.0691 (M^+^+1), found 487.0693.

*{5-[2-(4-Bromophenyl)-6-methoxyquinolin-4-yl}-N-(4-nitrophenyl)-[1,3,4]oxadiazol-2-amine* (**6i**). Yield 45%, m.p. 272–274 °C. ^1^H-NMR (DMSO-*d*_6_): 3.99 (s, 3H, OCH_3_), 7.55 (dd, 1H, *J* = 9.0, ArCH), 7.64 (d, 2H, *J* = 9.0, ArCH), 7.78 (d, 2H, *J* = 9.0, ArCH), 7.96 (s, 1H, ArCH), 8.10 (d, 3H, *J* = 9.2, ArCH), 8.24 (d, 2H, *J* = 8.6, ArCH), 8.39 (s, 1H, NH), 8.79 (d, 1H, *J* = 3.1, ArCH). HRMS (C_24_H_16_BrN_5_O_4_) *m*/*z* (ES^+^) required 517.0380 (M^+^+1), found 517.0428. Anal. (C_24_H_16_BrN_5_O_4_): % CHN required: C 56.10, H 3.27; found C 55.90, H 3.11.

*{5-[2-(4-Bromophenyl)-6-methoxyquinolin-4-yl}-N-(4-methoxyphenyl)-[1,3,4]oxadiazol-2-amine* (**6j**). Yield 57%, m.p. 232–234 °C. ^1^H-NMR (DMSO-*d*_6_): 3.69 (s, 3H, OCH_3_), 3.95 (s, 3H, OCH_3_), 6.90 (d, 2H, *J* = 7.3, ArCH), 7.58 (m, 3H, ArCH), 7.80 (d, 2H, *J* = 7.9, ArCH), 8.10 (d, 1H, *J* = 7.3, ArCH), 8.19 (d, 2H, *J* = 7.9, ArCH), 8.40 (s, 1H, ArCH), 8.54 (d, 1H, *J* = 2.5, ArCH). *m*/*z* (ES^+^) 505.07 (M^+^). Anal. (C_25_H_19_BrN_4_O_3_): % CHN required: C 59.65, H 3.80, N 11.13; found C 59.35, H 3.68, N 10.91.

*{5-[2-(4-Bromophenyl)-6-methoxyquinolin-4-yl}-N-(4-fluorophenyl)-[1,3,4]oxadiazol-2-amine* (**6k**). Yield 47%, m.p. 230–232 °C. ^1^H-NMR (DMSO-*d*_6_): 3.98 (s, 3H, OCH_3_), 7.12 (m, 2H, ArCH), 7.54 (d, 1H, *J* = 8.5, ArCH), 7.62 (m, 2H, ArCH), 7.78 (d, 2H, *J* = 8.5, ArCH), 8.08 (d, 1H, *J* = 8.5, ArCH), 8.19 (d, 2H, *J* = 7.8, ArCH), 8.31 (s, 1H, ArCH), 8.75 (d, 1H, *J* = 2.9, ArCH). *m*/*z* (ES^+^) 492.07 (M^+^). Anal. (C_24_H_16_BrFN_4_O_2_): % CHN required: C 59.01, H 3.68, N 10.90; found C 58.67, H 3.28, N 11.20.

### 4.5. Preparation of 5-[2-(4-Bromophenyl)-6-methoxyquinolin-4-yl]-4-aryl-4H-[1,2,4]triazole-3-thiols (**7a–b**).

A solution of 2-(2-(4-bromophenyl)-6-methoxyquinoline-4-carbonyl)-*N*-(4-aryl)hydrazine-1-carbothioamide (10 mmol) in 2N NaOH was heated under reflux for 3 h. After cooling to room temperature, water was added and the mixture carefully neutralized with 0.1M HCl. The precipitate formed was filtered, dried and recrystallized from ethanol to give the corresponding triazole thiol product (**7a–b**).

*5-[2-(4-Bromophenyl)-6-methoxyquinolin-4-yl]-4-(4-methoxyphenyl)-4H-[1,2,4]triazole-3-thiol* (**7a**). Yield 58%, m.p. 199–201 °C. ^1^H-NMR (DMSO-*d*_6_): 3.70 (s, 3H, OCH_3_), 3.87 (s, 3H, OCH_3_), 6.86 (d, 2H, *J* = 8.9, ArCH), 7.14 (d, 2H, *J* = 8.9, ArCH), 7.41 (dd, 1H, *J* = 9.3, ArCH), 7.51 (s, 1H, ArCH), 7.65 (d, 2H, *J* = 8.5, ArCH), 7.83 (m, 3H, ArCH), 7.95 (d, 1H, *J* = 9.2, ArCH).

*5-[2-(4-Bromophenyl)-6-methoxyquinolin-4-yl]-4-(3,4-dichlorophenyl)-4H-[1,2,4]triazole-3-thiol* (**7b**). Yield 64%, m.p. 170–172 °C. ^1^H-NMR (DMSO-*d*_6_): 3.86 (s, 3H, OCH_3_), 7.08 (dd, 1H, *J* = 8.3, ArCH), 7.42 (dd, 2H, *J* = 9.2, ArCH), 7.49 (d, 1H, *J* = 8.3, ArCH), 7.61 (d, 1H, *J* = 2.6, ArCH), 7.67 (d, 2H, *J* = 8.8, ArCH), 7.82 (d, 1H, *J* = 2.4, ArCH), 7.93 (d, 2H, *J* = 8.8, ArCH), 7.97 (d, 1H, *J* = 9.4, ArCH).

### 4.6. Preparation of 5-[2-(4-Bromophenyl)-6-methoxyquinolin-4-yl]-4-aryl-4H-[1,2,4]triazole-3-ylsulfanyl]-1-phenylethanone (**8a**–**b**). 

2-Bromoacetophenone (5 mmol) was added to a solution of 5-[2-(4-bromophenyl)-6-methoxyquinolin-4-yl]-4-aryl-4*H*-[1,2,4]triazine-3-thiol (**7a–b**, 5 mmol) in ethanol (70%) containing KOH (5 mmol). The reaction mixture was left to stir at room temperature for 16 h. Water was then added and the precipitate formed was filtered, dried and recrystallized from ethanol to give the corresponding S-alkyl triazole thiol product (**8a–b**).

*5-[2-(4-Bromophenyl)-6-methoxyquinolin-4-yl]-4-(4-methoxyphenyl)-4H-[1,2,4]triazole-3-ylsulfanyl]-1-phenylethanone* (**8a**). Yield 63%, m.p. 240–242 °C. ^1^H-NMR (DMSO-*d*_6_): 3.70 (s, 3H, OCH_3_), 3.87 (s, 3H, OCH_3_), 5.42 (s, 2H, CH_2_), 6.86 (d, 2H, *J* = 8.9, ArCH), 7.14 (d, 1H, *J* = 8.9, ArCH), 7.46 (m, 5H, ArCH), 7.51 (m, 2H, ArCH), 7.62 (d, 2H, *J* = 9.5, ArCH), 7.83 (m, 2H, ArCH), 8.15 (m, 3H, ArCH). *m*/*z* (ES^+^) 639.08 (M^+^).

*5-[2-(4-Bromophenyl)-6-methoxyquinolin-4-yl]-4-(3,4-dichlorophenyl)-4H-[1,2,4]triazole-3-ylsulfanyl]-1-phenylethanone* (**8b**). Yield 63%, m.p. 247–249 °C. ^1^H-NMR (DMSO-*d*_6_): 3.70 (s, 3H, OCH_3_), 5.12 (s, 2H, CH_2_), 7.32 (d, 1H, *J* = 2.3, ArCH), 7.54 (m, 2H, ArCH), 7.62 (m, 2H, ArCH), 7.76 (m, 4H, ArCH), 8.10 (m, 6H, ArCH), 8.16 (s, 1H, ArCH). Anal. (C_32_H_21_BrCl_2_N_4_O_2_S): % CHN required: C 56.82, H 3.13, N 8.28; found C 57.31, H 3.03, N 8.43.

### 4.7. Cell Viability—MTT Assay (MDA-MB-231 and HeLa Cells)

Human breast cancer MDA-MB-231 cells were cultured in RPMI 1640 medium (Life Technologies, Paisley, UK) supplemented with 10% foetal bovine serum, 100 IU/mL pencillin, and 100 μg/mL streptomycin (Life Technologies). Human cervical cancer HeLa cells were cultured in D-MEM (Life Technologies) supplemented with 10% foetal bovine serum, 100 IU/mL pencillin, and 100 μg/mL streptomycin (Life Technologies). Cells were passaged routinely and maintained at 37 °C and 5% CO_2_. For each experiment, 3000 cells in 0.2 mL of medium were seeded into each well of a clear flat-bottomed 96-well plate and allowed to adhere for 24 h. The cells were then incubated with test compound (from 10 mM stock in DMSO) over a 10 fold dilution series with concentrations ranging from 0.00001 mM to 100 μM, each diluent being performed in triplicate. Control experiments were conducted using DMSO vehicle control with volumes equivalent to test compound concentrations. Cells were incubated with test compound for 72 h., followed by treatment of wells with 20 μL of 3-[4,5-dimethylthiazol-2-yl]-2,5-diphenyl tetrazolium bromide (MTT) solution (from a freshly made 5.5 μg/mL stock in PBS, Sigma-Aldrich, Gillingham, Dorset, UK). After 4 hours the solutions from the wells were removed and the resulting formazan crystals were dissolved in DMSO (200 μL) and incubated for 30 minutes at 37 °C. Absorbance was measured at 550 nm using an automated microplate reader. Mean IC_50_ values were obtained from plots of absorbance versus test compound concentration using GraphPad Prism 5 software (San Diego, CA, USA). For each concentration, three independent repeat experiments were carried out to establish reproducibility.

### 4.8. Cell Viability—CellTiter-Blue® Assay (KG1a and Jurkat Cells)

Human acute myeloid leukaemia KG1a cells and acute T-cell lymphocytic Jurkat cells were cultured in RPMI 1640 medium (Life Technologies, Paisley, UK) supplemented with 10% foetal bovine serum, 100 IU/mL pencillin, and 100 μg/mL streptomycin (Life Technologies). Cells were passaged routinely and maintained at 37 °C and 5% CO_2_. For each experiment 15,000 cells in 0.1 mL of medium were seeded into each well of a solid black fluorescence 96-well plate and incubated for 24 h. The cells were then incubated with test compound (from 10 mM stock in DMSO) over a 10-fold dilution series with concentrations ranging from 0.00001 mM to 100 μM, each diluent being performed in triplicate. Control experiments were conducted using DMSO vehicle control with volumes equivalent to test compound concentrations. Cells were incubated with test compound for 24 h, followed by treatment with 20 μL of CellTiter-Blue® (Promega, Southampton, UK). Fluorescence was measured using 544 nm excitation and 590 nm emission filters on a fluorescent plate reader. Mean IC_50_ values were obtained from plots of absorbance versus test compound concentration using GraphPad Prism 5 software (San Diego, CA, USA). For each concentration, three independent repeat experiments were carried out to establish reproducibility.

### 4.9. Enzyme-Linked Immunosorbent Assay (ELISA)

A streptavidin-coated 96-well microtitre plate (Thermo Scientific, Rockford, IL) was washed three times with PBS containing 0.05% Tween-20. Biotinylated Bim peptide (residues 81–106) was diluted to 0.09 μg/mL (20 nM) in Superblock blocking buffer in PBS (Thermo Scientific), and aliquots (200 μL) were transferred to each well. Following incubation (1.5 h) at room temperature to allow immobilization of the Bim peptide on to the solid surface (via streptavidin-biotin interaction), the plate was washed three times with 0.5% BSA in PBS containing 0.05% Tween-20. Test compounds were dissolved in DMSO to obtain a 50 mM stock solution, and different concentrations of tested compound (10 fold dilution series from stock solution) were incubated with 20 nM His-tagged Bcl-2 protein (Abcam, Cambridge, UK) in PBS for one hour. The inhibitor and protein mixture (100 μL) were then transferred to the wells containing bound Bim peptide and incubated at room temperature for two hours. Following further washing of the plate in triplicate (0.5% BSA in PBS containing 0.05% Tween-20), anti-His antibody containing horseradish peroxidase enzyme (Qiagen, Crawley, UK) was diluted in 0.5% BSA in PBS (1:1000), and aliquots of 100 μL were added to each well. After incubation (one hour) at room temperature, the plate was washed three times as previously to remove any unbound antibody. A 200 μM solution of *o*-phenylenediamine (Sigma-Aldrich, UK) was freshly prepared at pH5 using phosphate-citrate buffer (Sigma-Aldrich), and hydrogen peroxide was added to the solution to give a final concentration of 0.004 %. Aliquots (200 μL) of the prepared solution were added to the wells and the plate incubated in the dark for 30 minutes at room temperature. The optical density was then read using a plate reader at 450 nm. The experiments were carried on three separate occasions, including both negative and positive controls, where the negative control contains all the components except the Bcl-2 protein, whereas the positive control contains all the components except the inhibitors.

The reduction of affinity of the Bim peptide for Bcl-2 was calculated as:

% Reduction in Bim affinity = l_450_ of treated wells (with inhibitor)/l_450_ of positive control × 100

A plot of log μM concentration for each inhibitor against the percentage reduction in affinity for the Bim peptide was created using non-linear regression curve analysis (GraphPad Prism 5), and the software used to generate the IC_50_ value for the Bcl-2 inhibition.

### 4.10. Molecular Modeling and Docking

All molecular modeling studies were performed on a RM Innovator Pentium IV (2.4 GHz) running Linux Fedora Core 3. The protein crystal structure of Bcl-2 was downloaded from (http://www.rcsb.org/pdb code: 4AQ3). Hydrogen atoms were added to the protein, using the protonate 3D option in MOE (Molecular Operating Environment). Ligand structures were built within MOE and energy minimized using the MMFF94x force field until a RMSD gradient of 0.05 Kcal mol^-1^ A°^-1^ was reached. The defined pocket was taken as the active site; Alpha triangle was chosen as the replacement methodology, London dG as the scoring function and ten conformations were retained for each compound.

## Figures and Tables

**Table 1:** Growth inhibitory activity (IC_50_, μM) values for quinolin-4-yl-*N*-aryloxadiazol-2-amines (**6a–k**) and quinolin-4-yl-benzoylmethylthiotriazoles (**8a–b**) in human cancer cell lines MDA-MB-231 (breast), HeLa (cervical), KG1a (AML) and Jurkat (T-cell leukaemia).

Compound	R	R ^1^	IC_50_ (μM) ^1^			
			MDA-MB-231 ^2^	HeLa ^2^	KG1a ^3^	Jurkat ^3^
**6a**	H	4-Cl-Ph	9.13 ± 0.82	7.13 ± 0.63	14.95 ± 0.54	44.25 ± 2.12
**6b**	H	3,4-diCl-Ph	0.25 ± 0.022	8.60 ± 0.72	70.52 ± 2.5	>100
**6c**	H	4-Me-Ph	0.55 ± 0.042	0.05 ± 0.002	16.99 ± 0.14	>100
**6d**	H	4-NO_2_-Ph	7.04 ± 0.11	6.39 ± 0.12	1.24 ± 0.13	56 ± 1.24
**6e**	H	4-OMe-Ph	9.85 ± 0.82	0.74 ± 0.04	1.31 ± 0.1	>100
**6f**	OMe	4-Cl-Ph	0.55 ± 0.01	0.84 ± 0.03	12.4 ± 0.27	>100
**6g**	OMe	3,4-diCl-Ph	0.34 ± 0.07	0.21 ± 0.04	26.96 ± 1.41	>100
**6h**	OMe	4-Me-Ph	0.43 ± 0.01	0.32 ± 0.021	12.25 ± 1.12	>100
**6i**	OMe	4-NO_2_-Ph	0.54 ± 0.02	1.42 ± 0.78	1.21 ± 0.044	>100
**6j**	OMe	4-OMe-Ph	0.44 ± 0.04	0.77 ± 0.09	0.36 ± 0.024	65 ± 1.47
**6k**	OMe	4-F-Ph	0.30 ± 0.01	5.44 ± 0.32	>100	>100
**8a**	-	4-OMe-Ph	>100	>100	>100	>100
**8b**	-	3,4-diCl-Ph	>100	>100	>100	>100

^1^ Mean of three independent experiments with range quoted; ^2^ MTT assay; ^3^ CellTitre-Blue**^®^** assay.

**Table 2:** Enzyme-linked immunosorbent assay (ELISA) binding activity values (IC_50_) for selected compounds compared to positive control (-)-gossypol. Results are expressed as triplicate testing mean values.

Compound	ELISA IC_50_ (μM) ^1^
**6c**	1.93 ± 0.007
**6d**	14.52 ± 1.20
**6i**	0.15 ± 0.02
**Gossypol**	0.60 ± 0.09

^1^ Mean of three independent experiments with range quoted.
